# Quality of Life of Patients Treated for Abdominal Aortic Aneurysm: Open Surgery and Endoprosthesis

**DOI:** 10.3390/jcm11082195

**Published:** 2022-04-14

**Authors:** Silvestra Barrena-Blázquez, Manuel Díez-Alonso, Luis Felipe Riera del Moral, Salvador Sanchez Coll, Melchor Alvarez-Mon, Miguel A. Ortega, Fernando Ruiz Grande

**Affiliations:** 1 Department of General Surgery, Príncipe de Asturias Hospital, 28801 Alcalá de Henares, Spain; manuelmariano.diez@salud.madrid.org; 2 Department of Surgery, Medical and Social Sciences, Faculty of Medicine and Health Sciences, University of Alcalá, 28801 Alcalá de Henares, Spain; 3 Department of Vascular Surgery, Nuestra Señora del Rosario Hospital, 28834 Madrid, Spain; luis.riera@salud.madrid.org (L.F.R.d.M.); ssanchezc@telefonica.net (S.S.C.); fruizgrande@hotmail.com (F.R.G.); 4 Department of Medicine and Medical Specialities, Faculty of Medicine and Health Sciences, University of Alcalá, 28801 Alcalá de Henares, Spain; mademons@gmail.com (M.A.-M.); miguel.angel.ortega92@gmail.com (M.A.O.); 5 Ramón y Cajal Institute of Sanitary Research (IRYCIS), 28034 Madrid, Spain; 6 Immune System Diseases-Rheumatology and Internal Medicine Service, University Hospital Príncipe de Asturias, (CIBEREHD), 28806 Alcalá de Henares, Spain; 7 Department of Vascular Surgery, Príncesa Hospital, 28834 Madrid, Spain

**Keywords:** abdominal aortic aneurysm, open abdominal repair, EVAR, SF-36 questionnaire, health-related quality of life

## Abstract

Objectives: To determine the degree of long-term health-related quality of life (HRQoL) of patients undergoing surgery for abdominal aortic aneurysm (AAA) and to analyze the results according to the type of treatment, namely, open abdominal repair (OAR) or endoprosthesis (EVAR). Patients and Methods: This was a prospective cross-sectional observational study. Patients receiving intervention for AAA between January 2013 and December 2020 were included. The Spanish version of the SF-36 questionnaire was used. A single survey was performed on all patients, and the time elapsed since the intervention was recorded. Results: On all health scales and in the two groups of patients, the highest scores were recorded at six months postoperatively. At that time, the EVAR and OAR groups had similar values. Between 13 and 16 months postoperatively, EVAR patients presented a transient but significant decrease in their scores for physical function (*p* = 0.016), vitality (*p* = 0.035) and social function (*p* = 0.041). From that moment, there were progressive decreases in the scores of the two groups of patients on all the scales of the SF-36 questionnaire, although this trend was less pronounced in the OAR group. At 60 months after the intervention, the latter group showed significantly higher values than EVAR for physical function (*p* = 0.01), vitality (*p* = 0.032) and mental health (*p* = 0.029). Additionally, at 60 months after the intervention, the Sum of the psychological component (MCS) and Sum of the physical component (PCS) scores were significantly higher in the OAR group (*p* = 0.040 and *p* = 0.039, respectively). Conclusions: In the short term, patients treated for AAA by EVAR or OAR showed similar results on the SF-36 questionnaire. In the long term, patients treated by EVAR had lower scores on the physical function, vitality and mental health scales.

## 1. Introduction

Abdominal aortic aneurysms (AAAs) are a major public health problem. Its prevalence in males is 1.3% between 45 and 54 years of age and rises to 12.5% between 75 and 84 years of age, with a male–female ratio of 4:1 [1]. The prevalence has increased in parallel with the increase in life expectancy and the decrease in cardiovascular mortality. The most serious complication is rupture, which usually requires urgent surgical intervention and is associated with a mortality rate close to 90% [2].

There are several options for the treatment of AAA: observation with medical treatment of cardiovascular comorbidities, open surgery with replacement by a prosthesis (open abdominal repair (OAR)) and the implantation of an endoprosthesis (EVAR) [3]. The type of treatment is selected based on the size and morphology of the aneurysm and the age and comorbidities of the patient. The morbidity and mortality rates of OAR are between 3 and 5%, and OAR is usually indicated in patients with low-moderate surgical risk [2,4]. The initial morbidity and mortality of EVAR is lower (1–1.5%), although the incidences of reoperation and long-term complications are high [5,6].

Both the EVAR and OAR techniques are now fully established, with satisfactory results, and their long-term survival rates are similar [5]. In recent years, it has become evident that the objectives considered important for surgeons (perioperative morbidity, mortality, long-term survival) can differ substantially in relation to the perception that the patient has regarding his or her state of health after treatment [7,8]. Health-related quality of life (HRQoL) has become a prominent parameter used to evaluate the outcome of treatment and should be an aid to guide patient expectations and in decision-making about a therapeutic option [7,8]. There is information on short-term HRQoL after the intervention [9,10,11]. However, there are few publications on long-term results (>12 months), the data are inconclusive, and comparisons between series are difficult.

The objective of this study was to determine the long-term HRQoL of AAA patients who underwent surgery and to analyze the results according to the type of treatment.

## 2. Patients and Methods

### 2.1. Design

Prospective cross-sectional observational study. Patients who intervened for AAA were consecutively included between January 2013 and December 2020 at the Prince of Asturias University Hospital in Alcalá de Henares and at the Nuestra Señora del Rosario Hospital in Madrid. The patients were identified from the computerized database that was completed prospectively with the data of all patients operated on in the Vascular Surgery Unit throughout these years. The study was developed according to the principles of the Declaration of Helsinki. The study was approved by the Ethics Committee of the Prince of Asturias Hospital. The patients accepted their participation and signed the consent document after being informed.

Patients undergoing scheduled surgery were included. Patients with thoracic or thoracic-abdominal aneurysm, patients who had occlusive involvement of the aorto-iliac sector, lack of understanding of the Spanish language, and people unable to make decisions were excluded.

According to the protocol followed, surgery was recommended when the aneurysm measured >5 cm in transverse diameter in the thoraco-abdominal-pelvic CT image performed with contrast if an AAA growth of 1.5 times the reference value of the aortic diameter or if an expansion of >0.5 cm/year was documented, when it produced symptoms or showed symptoms of rupture. EVAR was indicated in patients older than 65 years, with high surgical risk and with favourable aneurysm morphology (distance to the exit of the renal artery greater than 1.5 cm and absence of angled shapes that would hinder the anchorage of the prostheses). Coated bifurcated stents were used and implanted through an inguinal access with radiological control. The OAR was carried out by means of a median or transverse laparotomy, and a Dacron or PTFE prosthesis was implanted. Aorto-aortic and aorto-bifemoral reconstructions were used. The criteria for the indication of performing one technique or another (EVAR/AAA) remained unchanged during the years included in this study.

The clinical and demographic data of each patient were recorded in a specific data log and entered into a computerized database designed for this study with the statistical program Microsoft Excel 2019 (v.19) R, to which only the members of the research team had access. At all times, the anonymity of the patients was maintained, and the identification data were replaced by an alphanumeric code, of which only the principal investigator was aware. Data on comorbidities, cardiovascular risk factors, aneurysm morphology, type of intervention, perioperative morbidity, mortality and length of hospital stay were collected.

### 2.2. Quality of Life Study

To determine HRQoL, the validated Spanish version of the SF-36 questionnaire was used [12]. All patients were contacted by telephone to briefly inform them of the study and invite them to participate. All of the patients contacted accepted the invitation, and the majority went to the hospital where an interview with the principal investigator was carried out, in which the object of the study was explained in detail, the information sheet was delivered and the informed consent form was signed. Patients who could not travel to the hospital received the questionnaire over the phone. All interviews were conducted between December 2019 and December 2020. A single survey was performed on all patients, and the time elapsed since the intervention was recorded.

The SF36 questionnaire consists of thirty-six items or short questions that assess eight dimensions or scales of HRQoL: body pain (two items), mental health (five items), vitality (four items), social function (two items), general health (five items), physical function (ten items), emotional role (three items), and physical role (four items). To process the information that the patient provides when answering the test questions, the scores obtained in each of the items are coded, aggregated and transformed on a scale from 0 (worst health status) to 100 (best health status) using the algorithms and indications of the scoring and interpretation manual of the questionnaire [13]. These eight health scales are grouped into two summary measures: mental health (vitality, social function, emotional role and mental health) and physical health (physical function, physical role, body pain and general health). The final value of each summary measure is 50% of the average of the sum of the mean values obtained in the four scales included in it.

### 2.3. Statistical Analysis

For each aspect of health included in the SF-36 test, the mean, median, percentiles (25, 50, 75), standard deviation, and proportion of individuals with the maximum (ceiling effect) and minimum (floor effect) score were calculated. The psychometric properties of the scales of the quality-of-life test were analyzed: proportion of nonresponses, reliability through the Cronbach alpha coefficient and the correlation of the items that make up each dimension/scale with its total score (Spearman correlation).

To analyze the categorical variables, the number of observations and percentages were determined, and the chi-square test was used to compare results. For continuous variables, the normality of their distribution was analyzed (Kolmogorov test), and the results are presented as the mean, median, standard deviation, and percentiles. For comparisons, Student’s *t*-test and the Kruskal–Wallis test were used. For the statistical analysis, the SPSS program (v.23) (IBM, Armonk, NY, USA) was used.

## 3. Results

During the study period, 178 patients were operated on for AAA: 11 (6.2%) women and 167 (93.8%) men. The mean age was 73 ± 7 years (median: 72 years, range: 94–48 years). Of these, 109 were operated on by OAR and 69 by EVAR. Table 1 shows the characteristics of the patients, comorbidities and risk factors. Between the intervention and the completion of this study, 18 patients (16.5%) died in the OAR group and 9 (13%) in the EVAR group, so the HRQoL questionnaire was presented to the 151 patients (93 with OAR and 58 with EVAR) who remained alive.

Table 2 shows the results of the health scales obtained, without taking into account the time elapsed since the intervention. The group of patients operated on by OAR showed a significantly higher score than the EVAR group on the physical function (*p* = 0.001), vitality (*p* = 0.003), general health (*p* = 0.037), social function (*p* = 0.023) and mental health (*p* = 0.006) scales.

Table 3, Table 4 and Table 5 show the results classified according to the time elapsed between the intervention and the interview. On all of the health scales and in the two groups of patients, the highest scores were recorded at six months postoperatively. At that time, the values were similar between EVAR and OAR. Subsequently, between 13 and 16 months postoperatively, EVAR patients presented a transient but significant decrease in the scores for physical function (*p* = 0.016), vitality (*p* = 0.035), and social function (*p* = 0.041). From that point on, the scores on all the scales in the two groups of patients showed progressive decreases, although this trend was less pronounced in the OAR group. At 60 months after the intervention, the OAR group had significantly higher values than the EVAR group for physical function (*p* = 0.01), vitality (*p* = 0.032) and mental health (*p* = 0.029).

**Figure 1:**
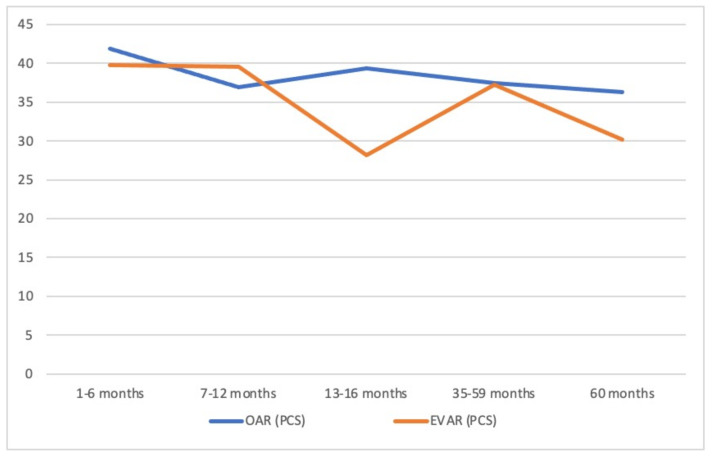
PCS scores according to the time elapsed from the intervention to the completion of the questionnaire for patients treated with OAR and EVAR (comparison at 60 months: *p* < 0.040). Sum of the physical component (PCS), open abdominal repair (OAR), endoprosthesis abdominal repair (EVAR).

**Figure 2:**
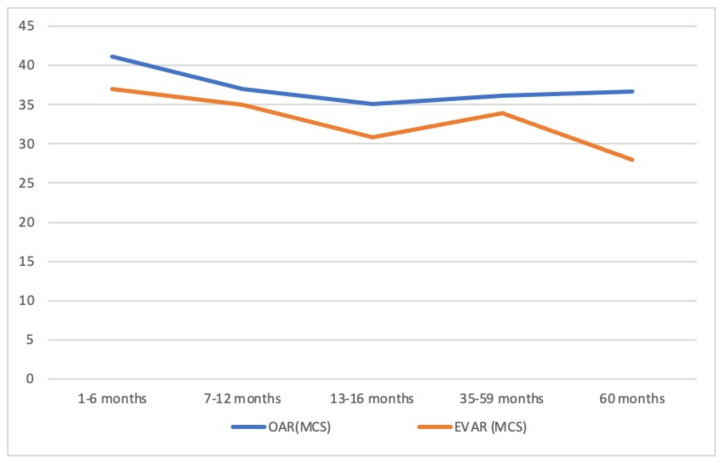
MCS scores according to the time elapsed from the intervention to the completion of the questionnaire for patients receiving intervention by OAR and EVAR (comparison at 60 months: *p* < 0.039). Open abdominal repair (OAR), endoprosthesis abdominal repair (EVAR), the Sum of the psychological component (MCS).

Figure 1 and Figure 2 show graphically the evolution of the two summary measures in the two groups of patients according to the time elapsed since the intervention and the interview. We observed that the PCS and MCS scores in the two groups of patients decreased with increases in the elapsed time. In the PSC, the significant decrease in the EVAR group stood out at 13–16 months postoperatively (*p* = 0.042). At 60 months, the PCS and MCS scores were significantly higher in the OAR group (*p* = 0.040 and *p* = 0.039, respectively).

## 4. Discussion

In recent years, attention has been given to the subjective perception of the patient about his or her physical and psychological well-being after treatment [7,8]. For patients, their symptoms are the main concern, regardless of whether the condition is serious [9]. The results of quality of life provide the basis for a holistic view of the patient and complement the traditional results of morbidity and mortality. In this study, we used the SF-36 test to measure HRQoL. This is the most widely used generic instrument in the international literature for this purpose in many fields of medicine [14].

In our study, the group of patients receiving intervention by OAR had significantly higher global scores for physical function and on the health scales included in the MCS (vitality, social function, emotional role, and mental health) than those with EVAR when the effect of the time elapsed since the intervention was not taken into account. However, the importance of these data, in isolation, is relative. The postoperative period of patients operated on for AAA is a dynamic process that goes through different stages, with different types of situations and potential complications [2,4]. In addition, one must consider the evolution of the underlying process must, usually atherosclerosis, and the natural deterioration that is associated with the passage of time.

We analyzed the influence of the time elapsed from the surgical intervention to the interview on HRQoL in the two groups of patients classified according to the treatment received. We observed the highest values on all scales of the SF-36 test at six months after surgery. The results obtained at that time were similar between patients with OAR and those with EVAR. The scores of the questionnaire obtained at that postoperative stage were similar to the reference values of the Spanish version in the adult population of the same age collected in a previous publication [15]. This indicates a good self-perception of health, perhaps due to the fact that the patients had already recovered from the impact of the intervention and since the number of postoperative complications in that time interval was low.

After six months, we recorded a progressive decrease in scores. However, the decrease was significantly lower in the OAR group for the physical function and on the scales of the MSC summary component (that is, the scores for mobility, personal care, return to usual activities and social integration were better in the OAR group). We found a drop in the scores observed between 13 and 16 months postoperatively in the EVAR group for physical function, vitality, and social function. We believe that this difference in HRQoL indicators is due to the higher incidence of late complications in the EVAR group. It is known that the most common complication, stent leakage, is more frequent at 12–18 months after the intervention [2,3,4]. In addition, it is known that from that period, other complications, such as aneurysm progression, occlusion at the outlet of the stent branches and thrombosis/ischemia of the lower limbs, are recorded in patients with EVAR. This requires a closer follow-up and successive reoperations in these patients [2,16]. For the patient, all of this represents a permanent unknown about their future evolution.

The data published in other studies show a significant decrease in the scores of all the scales of the SF-36 questionnaire in the first two months after the intervention and that this effect is greater in the group of patients with OAR, particularly on the scales for body pain and physical function [17,18,19]. This decrease is attributable to the overall impact of performing a laparotomy and a longer hospital stay. The data published in several randomized clinical trials and meta-analyses agree that after this first period, a recovery is recorded up to six months, with values similar to those before the intervention [19,20,21,22]. EVAR patients recovered to preoperative values more quickly, although as we verified in our series, at six months after the intervention, both groups showed similar scores on all scales.

In the medium term (6–12 months), both procedures show similar and coincident SF-36 scores with those of the general population of the same age [11,16,20,23].

However, few publications have analyzed the results of HRQoL beyond the first 12 months postoperatively. All of them have a transversal design, as in our work. We agree with the results obtained in the DREAM study in that in the long term, patients with OAR show better indicators of HRQoL. In this study, patients undergoing OAR and EVAR had similar results at six months postoperatively [24]. However, at 60 months of follow-up, the OAR group had higher scores on all scales and significantly higher scores on the scales encompassed in the MCS [25]. In the work by Peach [9], the scores provided by the patients in the EVAR group progressively decreased 12 months after surgery. Social life, perception of general health, concern about the future, and physical discomfort were the factors that most influenced the deterioration of the test. Additionally, Jean-Baptiste [26] found that in the long term, patients with OAR had higher scores on the SF-36 test and related the worse result obtained in patients in the EVAR group to the appearance of claudication in the lower limbs. In the study by Yildirim [27], the scores of the EVAR group on the scales of physical role, body pain, vitality, social function and general health were lower than those of the general population. Sexual function seems to be affected equally negatively in both groups, which can affect patient expectations [28] and is reflected in the scores on the MCS scales. It is known that the age of the patients is a factor that influences the final results of HRQoL [15]. There is a tendency to detect lower scores in the SF-36 test as the age of the patients increases. The mean age of the patients in the EVAR group was slightly higher than that in the RAA group (76 vs. 71, respectively), although the difference was not statistically significant (*p* = 0.09).

In contrast, other publications did not find differences in the OAR or EVAR scores. In fact, Hinterseher [29] found that after a mean follow-up of 67 months, the group of OAR patients had SF-36 values similar to those of the general German population, while the EVAR group had higher scores for vitality and mental health than the general population. For his part, Dick [30] found no differences between OAR and EVAR after a mean follow-up of 58 months.

Although it is difficult to compare the studies, it seems that the available information indicates that patients in the OAR group have a better long-term HRQoL, and that this is fundamentally based on a higher score on the scales of the MCS component.

The SF-36 questionnaire is the most commonly used test to evaluate HRQoL. It is a generic test. It is not specific to a specific pathology and has been used in very diverse processes. The validity of the results it provides is proven, and its acceptance is very broad. Its generic design allows an objective quantification of the HRQoL that facilitates the description of results and the comparison with other series and the values detected in control groups, representative of the general population of each country. Its main problem is its relative complexity in processing the raw data provided by the patient. Therefore, simplified derivations of the test have been described, such as the SF-8, which seems to provide comparable results [31]. On the other hand, it has been reported that since it is a nonspecific test, it can lose information that could be captured by tests designed for specific pathologies. In the field of AAA, specific tests have been described that allow obtaining qualitative information on the degree of HRQoL [9,32]. However, their value has not been verified, and they have greater complexity in terms of application and interpretation of results.

The main limitation of our study is that the collection of information from patients was carried out in a cross-sectional manner. Undoubtedly, these types of designs are not capable of capturing all the information on the patient’s progress. It would be very timely to carry out a randomized study with longitudinal development in the collection of information. On the other hand, our assignment of the type of treatment was not carried out randomly. This limitation may be alleviated by the uniformity of criteria in treatment and follow-up. One of the main limitations of our study is that the aspect of the age difference between both groups should be highlighted. There is significant evidence that there is an annual decline in QoL above 68 years [33], thus a 5-year age difference (71 years vs. 76 years) between the two groups could explain the deterioration in QoL in patients treated with EVAR.

## 5. Conclusions

The conclusion that we can obtain in this study is that, in the short term, patients treated for AAA by EVAR and OAR have similar results in the SF-36 test, although, in the long term, patients treated by EVAR have lower scores for physical function and on the scales included in the MSC summary component.

## Figures and Tables

**Table 1:** Characteristics of the patients receiving intervention and associated pathology. Open abdominal repair (OAR), endoprosthesis abdominal repair (EVAR), cerebral vascular accident (CVA), chronic obstructive pulmonary disease (COPD).

	OAR (*n* = 93)	EVAR (*n* = 58)
Age (mean ± SD)	71 (7)	76 (7)
Diabetes	16 (17.4%)	7 (12%)
Intermittent claudication	3 (3.2%)	1 (1.7%)
Lerich’s syndrome	1 (1%)	0 (0%)
ischemic heart disease	11 (11.8%)	11 (15.9%)
CVA	2 (2.1%)	5 (7.2%)
COPD	9 (9.6%)	8 (11.5%)
chronic renal failure	16 (17%)	7 (10.1%)
hyperuricemia	5 (5.3%)	0 (0%)
hypothyroidism	4 (4.6%)	2 (2.8%)
prostate pathology	16 (17.2%)	7 (10.1%)
SIZE OF THE ANEURISM		
40–56 mm	41 (44%)	30 (43.8%)
57–73 mm	28 (30%)	38 (55%)
73–90 mm	10 (10.7%)	1 (1.6%)
>90 mm	14 (15%)	0 (0%)
LOCATION		
Juxta-pararenal	15 (16%)	0 (0%)
Infrarenals	78 (84%)	69 (100%)
TYPE OF DERIVATION		
Bi-iliac aorto	47 (50.5%)	69 (100%)
Aorto-aortic	31 (33.5%)	0 (0%)
Bi-iliac-femoral aorto	8 (8.7%)	0 (0%)
Aorto-bifemoral	7 (7.2%)	0 (0%)
HOSPITAL STAY days (average)	10 (7)	4.14 (4)

**Table 2:** Global scores obtained on the health scales of the SF-36 Questionnaire according to the type of surgical intervention performed. Open abdominal repair (OAR), endoprosthesis abdominal repair (EVAR).

		Physical Function	Physical Role	Body Ache	General Health	Vitality	Social Function	Role Emotional	Mental Health
OAR (*n* = 93)	Media DS Median (range)	80.05 21.23 85 (100)	74.46 37.76 100 (100)	82.47 22.58 90 (100)	63.01 20.82 65 (85)	67.90 22.20 75 (100)	81.99 25.76 100 (100)	58.42 16.04 66.67 (100)	75.48 23.46 80 (80)
EVAR (*n* = 58)	Media DS Median (range)	69.05 22.48 75 (100)	71.55 38.74 100 (100)	80.52 24.95 90 (100)	56.47 14.23 57.5 (55)	58.62 18.77 60 (80)	75.65 25.15 87.5 (100)	59.77 14.98 66.67 (67)	68.83 18.21 72 (80)
*p* value		0.001	0.623	0.704	0.037	0.003	0.023	0.562	0.006

**Table 3:** Scores obtained on the health scales of the SF-36 Questionnaire in the OAR group of patients according to the time elapsed since the intervention. Open abdominal repair (OAR), abdominal repair (AR).

		Physical Function	Physical Role	Body Ache	General Health	Vitality	Social Function	Role Emotional	Mental Health
1–6 months	AR (*n* = 14) Mean ± SD Median (range)	87.14 ± 8.25 85 (30)	87.5 ± 21.3 100 (100)	87.86 ± 13.6 90 (40)	73.93 ± 14.3 75 (50)	82.5 ± 8.93 85 (30)	96.43 ± 7.6 100 (25)	64.29 ± 8.9 66.6 (50)	86 ± 11.39 88 (90)
7–12 months	AR (*n* = 12) Mean ± SD Median (range)	79.58 ± 23.3 87.5 (85)	72.9 ± 37.6 100 (100)	85.83 ± 18.8 95 (50)	62.08 ± 16.5 65 (50)	72.08 ± 16.7 75 (50)	84.38 ± 14.2 87.5(37)	58.33 ± 15.07 66.6 (34)	76.67 ± 12.39 78 (40)
13–16 months	AR (*n* = 27) Mean ± SD Median (range	77.04 ± 24 85 (100)	67.5 ± 43.1 100 (100)	79.26 ± 23.1 80 (70)	60.19 ± 22.7 65 (85)	62.22 ± 15.5 65 (85)	74.54 ± 10.7 87.5 (85)	54.32 ± 18.83 66.6 (50)	70.52 ± 27.26 80 (80)
35–59 months	AR (*n* = 20) Mean ± SD Median (range)	79.25 ± 21.8 85 (90)	70 ± 42.6 100 (100)	78 ± 26.2 95 (80)	62.5 ± 20.9 72.5 (65)	68.5 ± 22 75 (80)	88.13 ± 21.6 100 (75)	63.33 ± 14.9 66.6 (70)	79.8 ± 20.62 80 (80)
≥60 months	AR (*n* = 20) Mean ± SD Median (range)	80.25 ± 22.1 82.5 (95)	80 ± 34 100 (100)	85.5 ± 25.2 100 (100)	60.25 ± 23.3 62.5 (75)	69.25 ± 13.3 62.5 (70)	74.38 ± 31 87.5 (100)	55 ± 16.31 66 (34)	78.8 ± 19.57 82 (80)
*p* value		0.905	0.853	0.704	0.542	0.069	0.068	0.562	0.525

**Table 4:** Scores obtained on the health scales of the SF-36 Questionnaire in the EVAR group of patients according to the time elapsed since the intervention. Endoprosthesis abdominal repair (EVAR).

		Physical Function	Physical Role	Body Ache	General Health	Vitality	Social Function	Role Emotional	Mental Health
1–6 months	EVAR (*n* = 7) Mean ± SD Median (range)	82.14 ± 17.5 90 (50)	82.1 ± 37.4 100 (100)	87.14 ± 11.1 90 (20)	63.57 ± 11.8 70 (30)	72.86 ± 18.2 70 (50)	92.86 ± 6.6 87.5 (12)	61.9 ± 12.59 66.6 (45)	77.14 ± 14.18 80 (44)
7–12 months	EVAR (*n* = 6) Mean ± SD Median (range)	75 ± 20.9 70 (50)	95.8 ± 10.2 100 (100)	75 ± 27.3 80 (70)	65 ± 8.36 65 (25)	68.3 ± 16.9 72.5 (50)	85.42 ± 9.4 87.5 (25)	61.11 ± 13.6 66.6 (45)	69.33 ± 17.64 66 (44)
13–16 months	EVAR (*n* = 15) Mean ± SD Median (range)	62.67 ± 19.2 55 (65)	63.3 ± 44.1 100 (100)	75.3 ± 29.4 90 (90)	55 ± 14.2 55 (45)	52.6 ± 18.5 50 (60)	62.50 ± 27.1 62.5 (100)	53.33 ± 21.08 66.6 (67)	62.4 ± 23.26 64 (80)
35–59 months	EVAR (*n* = 14) Mean ± SD Median (range)	72.86 ± 17.9 75 (65)	75 ± 33.9 87.5 (100)	82.8 ± 28.4 100 (90)	54.64 ± 15.7 52.5 (45)	57.5 ± 18.5 57.5 (70)	86.61 ± 22.7 100 (62)	64.29 ± 8.9 66.67 (45)	72.86 ± 16.61 76 (60)
≥60 months	EVAR (*n* = 16) Mean ± SD Median (range)	63.75 ± 19.1 70 (95)	62.5 ± 42.8 87.5 (100)	82.5 ± 22 90 (70)	53.12 ± 14.7 50 (50)	53.3 ± 17.6 57.5 (60)	67.19 ± 26.1 75 (87)	60.42 ± 13.43 66.6 (45)	67.5 ± 15.51 72 (66)
*p* value		0.279	0.461	0.805	0.037	0.014	0.004	0.466	0.490

**Table 5:** Degree of significance of the comparisons of the health scales of the SF-36 Questionnaire at 6 and 60 months after the intervention in the EVAR and OAR groups (Kruskal–Wallis test). Open abdominal repair (OAR), endoprosthesis abdominal repair (EVAR).

	Physical Function	Physical Role	Body Ache	General Health	Vitality	Social Function	Role Emotional	Mental Health
6 months	0.03	0.219	0.431	0.962	0.539	0.961	0.697	0.32
16 months	0.016	0.769	0.859	0.286	0.035	0.041	0.950	0.226
60 months	0.023	0.246	0.420	0.296	0.032	0.122	0.439	0.029

## Data Availability

The data used to support the findings of the present study are available from the corresponding author upon request.
